# Diffusion Logarithm-Correntropy Algorithm for Parameter Estimation in Non-Stationary Environments over Sensor Networks

**DOI:** 10.3390/s18103381

**Published:** 2018-10-10

**Authors:** Limei Hu, Feng Chen, Shukai Duan, Lidan Wang

**Affiliations:** 1 College of Electronic and Information Engineering, School of Mathematics and Statistics, Southwest University, Chongqing 400715, China; hlm0903@email.swu.edu.cn; 2 Key Laboratory of Nonlinear Circuits and Intelligent Information Processing, and College of Electronic and Information Engineering, Southwest University, and Chongqing Collaborative Innovation Center for Brain Science, Chongqing 400715, China; duansk@swu.edu.cn (S.D.); ldwang@swu.edu.cn (L.W.)

**Keywords:** non-stationary, sensor networks, parameter estimation, diffusion logarithm-correntropy algorithm, tracking performance

## Abstract

This paper considers the parameter estimation problem under non-stationary environments in sensor networks. The unknown parameter vector is considered to be a time-varying sequence. To further promote estimation performance, this paper suggests a novel diffusion logarithm-correntropy algorithm for each node in the network. Such an algorithm can adopt both the logarithm operation and correntropy criterion to the estimation error. Moreover, if the error gets larger due to the non-stationary environments, the algorithm can respond immediately by taking relatively steeper steps. Thus, the proposed algorithm achieves smaller error in time. The tracking performance of the proposed logarithm-correntropy algorithm is analyzed. Finally, experiments verify the validity of the proposed algorithmic schemes, which are compared to other recent algorithms that have been proposed for parameter estimation.

## 1. Introduction

Sensor networks are useful tools for disaster relief management, target localization and tracking, and environment monitoring [1,2,3,4]. Distributed parameter estimation plays an essential role in sensor networks [5,6,7]. The objective of the parameter estimation is to estimate some essential parameters from noisy observation measurements through cooperation between nodes. Moreover, distributed strategies are of great significance to solve the problem of parameter estimation in sensor networks, due to their robustness against imperfections, low complexity, and low power demands.

Among these distributed schemes, in the incremental strategy [8], a cyclic path is defined over the nodes and data are processed in a cyclic manner through the network until optimization is achieved. However, determining a cyclic path that runs across all nodes is generally a challenging (NP-hard) task to perform. In the consensus strategy [9], vanishing step sizes are used to ensure that nodes can reach consensus and converge to the same optimizer in steady-state. In the diffusion strategy, information is processed locally and simultaneously at all nodes. The processed data are diffused through a real-time sharing mechanism that ripples through the network continuously [10,11]. The diffusion strategies are particularly attractive because they are robust [12,13,14,15], flexible, and fully distributed compared with incremental and consensus strategies, so we adopt diffusion strategies in this paper.

Most prior literature is mainly concerned with the case where nodes estimate the parameter vector collaboratively in the stationary case over sensor networks [10,16]. However, in the real world, the non-stationary case is normal. In this work, we mainly consider the parameter estimation in the non-stationary case, where the parameter is always time-varying. The observation data are nonlinear and non-Gaussian, since the data may be disturbed by changing communication links or outliers under the non-stationary environments.

Inspired by the differentiability and mathematical tractability of logarithm functions, we introduce the logarithm function as the error cost function [17]. Moreover, the correntropy criterion is a nonlinear measure of similarity between two random variables [18], which is a robust optimality criterion has been successfully used in the field of non-Gaussian signal processing. To make the error cost function more suitable for non-stationary environments, we propose a diffusion signal processing framework with a logarithm-correntropy cost function to solve the parameter estimation problem, which can elegantly and gradually adjust the cost function in its optimization based on the error amount.

A. Related Works 

The tracking behavior of a wide range of adaptive networks under non-stationary conditions was thoroughly investigated in [19,20,21,22]. In stationary conditions, based on the *p* norm error criterion, a diffusion minimum average p-power (dLMP) was proposed to estimate the parameters in wireless sensor networks [23]. To estimate the mean-square weight deviations under the zero-mean stationary measurement noise, the proportionate-type normalized least mean square algorithms were proposed in [24]. The diffusion normalized least-mean-square algorithm (dNLMS) was proposed for parameter estimation in a distributed network [25], and the variable step size of the dNLMS algorithm was obtained by minimizing the mean-square deviation to achieve fast convergence rate. The gradient-descent total least-squares (dTLS) algorithm is a stochastic-gradient adaptive filtering algorithm that compensates for error in both input and output data [26]. The steady-state analysis of gradient-descent total least-squares was inspired by the energy-conservation-based approach to the performance analysis of adaptive filters. When measurement noise involves impulsive interference, Ni, Chen, and Chen [27] designed a diffusion sign-error LMS (dSE-LMS) to solve the parameter estimation. The tracking performance of a variable step-size diffusion LMS algorithm is considered in non-stationary environment [28], but this research did not get the closed-form expression of steady-state mean-square deviation (MSD) or excess mean-square error (EMSE) of the network. Consequently, the theory and simulation do not match well. To date, the performance of distributed estimation algorithms has been predominantly studied under stationary conditions. However, the performances of these algorithms may degrade in non-stationary environments.

To find the optimal adaptation step sizes over the networks, Abdolee, Vakilian, and Champagne [29] formulated a constrained nonlinear optimization problem and solved it through a log-barrier Newton algorithm in an iterative manner. By using the optimal step size at each node, the performance of diffusion least-mean squares (DLMS) could be improved in non-stationary signal environments. Compared with this research, the proposed algorithm can respond immediately by taking relatively steeper steps when the error gets larger, and as a result, the new algorithm can perform well in non-stationary environments without finding the optimal step size at each node.

B. Our Contributions and Organization 

To further promote estimation performance in non-stationary environments over sensor networks, a novel algorithm needs to be designed. In this paper, the random-walk model is introduced for non-stationary environments. We proposed the logarithm-correntropy algorithm for parameter estimation in sensor networks under the non-stationary environments. This algorithm can adopt both the logarithm operation and correntropy criterion to the estimation error. Moreover, if the error gets larger due to the non-stationary environments, the algorithm can respond immediately by taking relatively steeper steps. Thus, the proposed algorithm achieves smaller error in time. The tracking performance of the proposed algorithm was analyzed. Simulation results are presented to evaluate the proposed algorithm.

The rest of this paper is organized as follows. In Section 2, we describe the estimation problem in a non-stationary environment. Section 3 introduces the adapt-then-combine (ATC) diffusion diffusion logarithmic-correntropy algorithm. In Section 4, the tracking performance analysis of the proposed algorithm is presented. Simulation results are presented in Section 5. Finally, conclusions are drawn in Section 6.

*Notation:* In what follows, let bold letters denote random variables and non-bold letters represent their realizations. Operators ${\left(.\right)}^{T}$ and $E\left[.\right]$ denote transposition and expectation, respectively. $I_{m}$ denotes an m×m identity matrix. 1 is an N×1 all-unity vector. $\left|.\right|$ is the absolute value of a scalar.

## 2. Estimation Problem in a Non-Stationary Environment

Consider a network with *N* nodes (sensors) deployed to observe some physical phenomena and specific events in a special environment. It is fundamentally necessary to consider and analyze parameter estimation under non-stationary conditions with the intent of employing them for practical applications. One challenge confronted in real-world applications is the non-stationary nature of the underlying parameters. For this purpose, a data model with a varying parameter is required. In this paper, we use the random walk model in [19] to depict the non-stationary condition.

**Assumption** **1.** *(Random Walk Model): The parameter vector varies based on the following model:*

(1) $$w_{i}^{*}=w_{{}_{i-1}}^{*}+\eta_{i},$$

*where $w_{i-1}^{*}$ is a random variable with a constant mean, where i is the time index. $\eta_{i}$ is a zero-mean random sequence with a covariance matrix $R_{\eta }$.*

**Assumption** **2.** *The sequence $\eta_{i}$ is independent of $u_{k,i}$ and $n_{k,i}$ for all k and i.*

**Assumption** **3.** *The initial conditions $w_{-1}^{*}$ are independent of all $d_{k,i}$ , $u_{k,i}$ , $n_{k,i}$, and $\eta_{i}$.*

At every time *i*, every node *k* can only exchange information with the nodes from its neighborhoods $N_{k}$ (including node *k* itself), and takes a scalar measurement $d_{k,i}$ according to:

(2) $$d_{k,i}={\left(w_{i}^{*}\right)}^{T}u_{k,i}+n_{k,i},$$

where $u_{k,i}$ denotes the M×1 random regression input signal vector and we assume I>M, $n_{k,i}$ is the Gaussian noise with zero mean and variance $\sigma_{n,k}^{2}$. The problem is to estimate an M×1 unknown varying vector $w_{i}^{*}$ at each node *k* from collected measurements. The objective of the network is to search for all unknown variable *w* and find the best estimation $w^{*}$ at the end by minimizing the MSE cost function in a distributed manner as follows:

(3) $$J_{k}\left(w\right)=E{\left|d_{k,i}-w^{T}u_{k,i}\right|}^{2}.$$

The cost function of the global network can be described as:

(4) $$\min_{\left(w\right)}\;\;\;\;\;J^{global}\left(w\right)=\sum_{k=1}^{N}E{\left|d_{k,i}-w^{T}u_{k,i}\right|}^{2}.$$

The optimization problem in Equation (3) can be solved by the diffusion strategies proposed in [30,31]. In these strategies, the estimate for each node is generated through a fixed combination strategy, which refers to giving different weights to the estimation of *k*’s neighbors to minimize the local function as follows:

(5) $$J_{k}^{local}\left(w\right)=\sum_{l\in N_{k}}c_{lk}E\left|d_{l,i}-w^{T}u_{l,i}\right|,$$

where $c_{lk}$ is the combination coefficient. For simplicity and good performance, we use the Metropolis rule in our work. The description of the Metropolis rule is:

(6) $$c_{l,k}=\left\{\begin{array}{c} \frac{1}{\max\left(n_{k,}n_{l}\right)},\;\mathrm{if}\;\;l\in N_{k}\backslash k,\\ 1-\sum_{l\in N_{k}\backslash k}c_{lk},\;\mathrm{if}\;\;l=k,\\ 0.\;\;\;\;\;\;\;\;\;\;\;\;\;\;\;\;\;\;\;\;\;\mathrm{if}\;l\notin N_{k}, \end{array}\right.$$

where $n_{k}$ is the degree of node *k* (the number of nodes connected to node *k*). The combining coefficients $c_{lk}$ also satisfy the following conditions: $\sum_{l\in N_{k}\cup k}c_{lk}=1$ and $c_{lk}=0\;\;if\;\;l\notin N_{k},\;CI=I,\;I^{T}C=I^{T}$, where *C* is an N×N matrix with non-negative real entries $\left\{c_{lk}\right\}$.

## 3. Diffusion Logarithmic-Correntropy Algorithm

In the non-stationary case, the parameter is always time-varying. We propose a new logarithmic-correntropy method to solve the parameter estimation problem. In order to solve Equation (3), since nodes in sensor networks have access to the observed data, we can take advantage of node cooperation by introducing a distributed diffusion learning manner.

In this paper, we are inspired from the recent developments in the information theoretic learning (ITL) related to the “logarithmic cost function” and the “correntropy”-based approaches [17,32]. The logarithmic function is differentiable, which makes it mathematically tractable. We introduce the logarithmic function as an efficient cost function in the adaptive algorithm. In this framework, we introduce an error cost function using the logarithmic function given by:

(7) $$J\left(e_{k,i}\right)=F\left(e_{k,i}\right)-\frac{1}{\alpha }\ln\left(1+\alpha F\left(e_{k,i}\right)\right),$$

where α>0 is a a small systemic parameter and $F\left(e_{l,i}\right)$ is a conventional cost function of the estimation error $e_{k,i}$ on each node *k*. The estimation error is $e_{k,i}=d_{k,i}-w^{T}{u_{k}}_{,i}$. In this paper, we introduce the correntropy criterion to formulate the conventional cost function $F\left(.\right)$. The correntropy is a similarity measure based on the ITL criterion. Given two random variables *X* and *Y*, the corresponding correntropy between them can be defined by [33]:

(8) $$V\left(X,Y\right)=E\left[k_{\sigma }\left(X-Y\right)\right]=\int k_{\sigma }\left(x-y\right)dF_{XY}\left(x,y\right),$$

where $k_{\sigma }\left(.\right)$ is a continuous, symmetric, positive-definite function with bandwidth σ, also called the Mercer kernel. *E* is an expectation operator. The joint distribution function of *X* and *Y* is $F_{XY}\left(x,y\right)$. The Gaussian kernel is mainly concerned in this paper.

(9) $$k_{\sigma }\left(x-y\right)=\exp\left(-\frac{{\left(x-y\right)}^{2}}{2\sigma^{2}}\right)$$

For each node *k*, based on the correntropy criterion, the instantaneous conventional cost function $F\left(e_{k,i}\right)$ is:

(10) $$F\left(e_{k,i}\right)=k_{\sigma }\left(e_{k,i}\right)=\exp\left(-\frac{{\left(d_{k,i}-w^{T}u_{k,i}\right)}^{2}}{2\sigma^{2}}\right)=\exp\left(-\frac{{\left(e_{k,i}\right)}^{2}}{2\sigma^{2}}\right).$$

In non-stationary conditions over networks, the communication among nodes is subject to link noise, and it is natural that the observation vectors are affected by noise. The total least squares (TLS) method for estimation can have desirable performance by reducing the noise effect from both the observation vector and the data matrix [34]. We briefly explain the TLS method as follows:

Consider the linear parameter estimation problem Ax≈b, where A is the data matrix, b is the observation vector, and x is the unknown parameter vector. The least squares (LS) approach considers that the observation vector is noisy while the data matrix is noiseless. However, the total least squares (TLS) approach considers that both the observation vector and the data matrix are noisy [35]. The LS approach seeks the estimate of the unknown parameter vector x by minimizing a sum of squared residuals expressed by:

(11) $$\min_{x}\;{\left(\mathrm{Ax}-b\right)}^{2},$$

while the TLS approach minimizes a sum of weighted squared residuals expressed by:

(12) $$\min_{x}\;\frac{{\left(\mathrm{Ax}-b\right)}^{2}}{{\left(x\right)}^{2}+1}.$$

From the matrix algebra viewpoint, the total least squares (TLS) approach is a refinement of the LS method when there are errors in both the observation vector and the data matrix. Inspired by the desirable features of the TLS method, to make the logarithm-correntropy method more suitable for non-stationary environments, we rewrite the conventional cost function as:

(13) $$\tilde{F}\left(e_{k,i}\right)=\exp\left(-\frac{{\left(d_{k,i}-w^{T}u_{k,i}\right)}^{2}}{2\sigma^{2}\left(w^{2}+1\right)}\right)=\exp\left(-\frac{{\left(e_{k,i}\right)}^{2}}{2\sigma^{2}\left(w^{2}+1\right)}\right).$$

**Figure 1:**
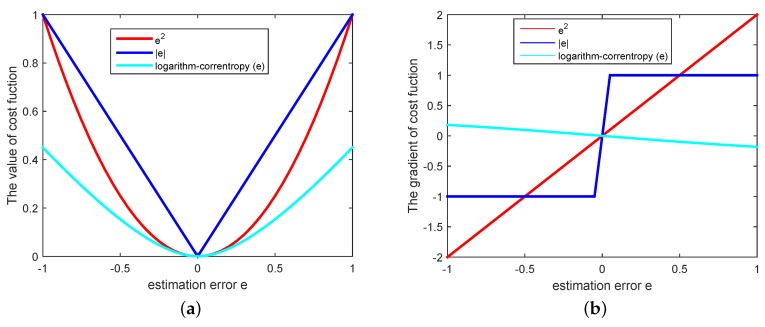
(**a**) The value of cost function; (**b**) The gradient of cost error function.

To demonstrate the superiority of the proposed logarithm-correntropy method, we introduce different stochastic cost functions, such as the least mean square cost $e^{2}$ and absolute difference cost $\left|e\right|$. Figure 1 compares these cost functions with the proposed cost function logarithm-correntropy $\left(e\right)$. It can be observed that the proposed cost function logarithm-correntropy $\left(e\right)$ is less sensitive to tiny interference on the error, and shows comparable steepness for quite large error interference. Furthermore, this new logarithm-correntropy cost function benefits from mapping the original input space into a potential higher-dimensional “feature space”, where linear methods can be employed. Particularly, if the error gets larger due to the non-stationary environments, the algorithm can respond immediately by taking relatively steeper steps. Thus, the proposed algorithm achieves smaller error in time and takes more gradual steps in space.

Given the data model, all nodes can observe data generated by the data model in Equation (2). It is natural to expect collaboration between nodes to be beneficial for a distributed sensor network. This means that neighbor nodes can share information with each other as permitted by the network topology. Therefore, according to Equations (7) and (13), we define the global cost function so that all nodes in the sensor network can be adapted in a distributed manner, then the new global function can be built as follows:

(14) $$\begin{array}{c} J^{global}\left(w\right)=\sum_{k=1}^{N_{k}}\left(\tilde{F}\left(e_{k,i}\right)-\frac{1}{\alpha }\ln\left(1+\alpha \tilde{F}\left(e_{k,i}\right)\right)\right)\\ =\sum_{k=1}^{N_{k}}\left(\exp\left(-\frac{{\left(e_{k,i}\right)}^{2}}{2\sigma^{2}\left(w^{2}+1\right)}\right)+\frac{1}{\alpha }\ln\left(1+\alpha \exp\left(-\frac{{\left(e_{k,i}\right)}^{2}}{2\sigma^{2}\left(w^{2}+1\right)}\right)\right)\right). \end{array}$$

To develop the distributed diffusion logarithm-correntropy algorithm in non-stationary environments over sensor networks, we can build the following new diffusion Logarithm-Correntropy Algorithm (dLCA) local cost function at every node *k* as:

(15) $$\begin{array}{c} J_{k}^{local}\left(w_{k,i}\right)=\sum_{l\in N_{k}}^{}c_{lk}\left(\tilde{F}\left(e_{l,i}\right)-\frac{1}{\alpha }\ln\left(1+\alpha \tilde{F}\left(e_{l,i}\right)\right)\right)\\ =\sum_{l\in N_{k}}c_{lk}H\left(e_{l,i}\right)\\ =\sum_{l\in N_{k}}c_{lk}\left(\exp\left(-\frac{{\left(e_{l,i}\right)}^{2}}{2\sigma^{2}\left({\left(w_{l,i}\right)}^{2}+1\right)}\right)+\frac{1}{\alpha }\ln\left(1+\alpha \exp\left(-\frac{{\left(e_{l,i}\right)}^{2}}{2\sigma^{2}\left({\left(w_{l,i}\right)}^{2}+1\right)}\right)\right)\right), \end{array}$$

where $w_{k,i}$ is the local estimate obtained by node *k* at time *i*, $e_{l,i}=d_{l,i}-{\left(w_{l,i}\right)}^{T}u_{l,i}$ is the estimation error at node *l*, *l* denotes any neighbor node of node *k*, $H\left(e_{l,i}\right)=\tilde{F}\left(e_{l,i}\right)-\frac{1}{\alpha }\ln\left(1+\alpha \tilde{F}\left(e_{l,i}\right)\right)$ , and the $c_{l,k}$ denote combining coefficients, which also is subjected to the Metropolis rule.

To reach the minimum $w_{i}^{*}$, it is a natural thought to use the steepest-descent method. Taking the derivative of Equation (15), we have

(16) $$\frac{\partial J^{local}\left(w_{k,i}\right)}{\partial w_{k,i}}=\sum_{l\in N_{k}}c_{lk}\frac{\partial H\left(e_{l,i}\right)}{\partial w_{l,i}}=\sum_{l\in N_{k}}c_{lk}\left(\tau_{l,i}+\frac{\tau_{l,i}}{1+\alpha \zeta_{l,i}}\right),$$

where $\xi_{l,i}=\exp\left(-\frac{{\left(e_{l,i}\right)}^{2}}{2\sigma^{2}\left({\left(w_{l,i-1}\right)}^{2}+1\right)}\right)$ and $\tau_{l,i=}\left(\exp\left(-\frac{{\left(e_{l,i}\right)}^{2}}{2\sigma^{2}\left({\left(w_{l,i-1}\right)}^{2}+1\right)}\right).\frac{e_{l,i}\left(u_{l,i}+d_{l,i}w_{l,i}\right)}{\sigma^{2}{\left({\left(w_{l,i-1}\right)}^{2}+1\right)}^{2}}\right)$.

**Figure 2:**
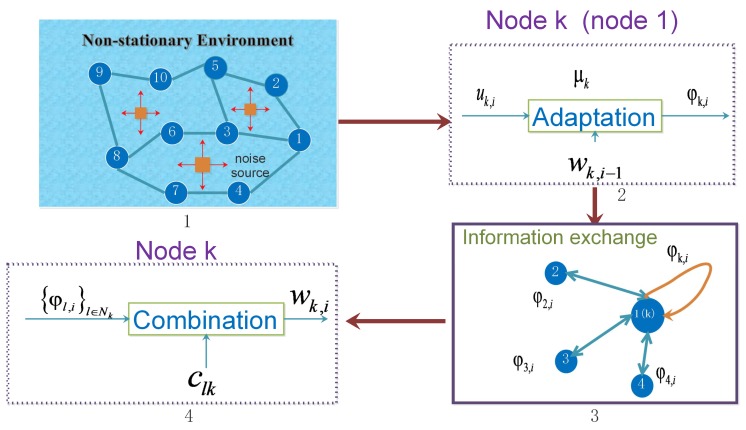
Adapt-then-Combine (ATC) diffusion strategies. Step 1 depicts a sensor network working in a non-stationary environment. In the adaptation stage 2, each node is using observed data $\left(u_{k,i},d_{k,i}\right)$ to update its intermediate estimate $\varphi_{k,i}$. Step 3 shows the information exchanging process between nodes. In the combination stage 4, each node collects the intermediate estimates from its neighbors.

Since nodes in the sensor networks have access to all observed data, we can take advantage of node cooperation by introducing a diffusion strategy to estimate the parameter $w_{k,i}$ in a fully distributed manner. This paper concerns the Adapt-then-Combine (ATC) scheme of the diffusion strategy. As Figure 2 shows, in the ATC scheme, nodes in networks combine information from their immediate neighbors firstly, and then employ updates by the following steps:

(1) Adaptation: In order to obtain an intermediate estimate, we introduce a step-size parameter μ. Each node updates its current estimate for the true parameter value by taking steepest-descent method. We can obtain an intermediate estimate $\varphi_{k,i}$ as follows:

(17) $$\varphi_{k,i}=\varphi_{k,i-1}-\mu \frac{\partial H\left(e_{k,i}\right)}{\partial w_{k,i-1}}=\varphi_{k,i-1}-\mu \left(\tau_{l,i}+\frac{\tau_{l,i}}{1+\alpha \xi_{l,i}}\right).$$

(2) Combination: This step is also called the diffusion step, to obtain a new estimate, each node aggregates its own intermediate estimate from all its neighbor nodes as follows:

(18) $$w_{k,i}=\sum_{l\in N_{k}}c_{lk}\varphi_{l,i}.$$

For the purpose of clarity, we summarize the procedures of the diffusion Logarithm-Correntropy Algorithm (dLCA) (Algorithm 1) as follows: 

**Algorithm 1:** diffusion Logarithm-Correntropy Algorithm   **Initialize:** Start with $\left\{w_{l,-1}=0\right\}$ for all *l*, initialize $w_{k,0}$ for each node   *k*,step-size μ, and cooperative coefficients $c_{lk}$. Set α>0, σ>0.   **for**
t=1:T
      **for each node *k*:**     **Adaptation**.        $\xi_{l,i}=\exp\left(-\frac{{\left(e_{l,i}\right)}^{2}}{2\sigma^{2}\left({\left(w_{l,i-1}\right)}^{2}+1\right)}\right)$        $\tau_{l,i=}\left(\exp\left(-\frac{{\left(e_{l,i}\right)}^{2}}{2\sigma^{2}\left({\left(w_{l,i-1}\right)}^{2}+1\right)}\right).\frac{e_{l,i}\left(u_{l,i}+d_{l,i}w_{l,i}\right)}{\sigma^{2}{\left({\left(w_{l,i-1}\right)}^{2}+1\right)}^{2}}\right)$.        $\varphi_{k,i}=\varphi_{k,i-1}-\mu \frac{\partial H\left(e_{k,i}\right)}{\partial w_{k,i-1}}=\varphi_{k,i-1}-\mu \left(\tau_{l,i}+\frac{\tau_{l,i}}{1+\alpha \xi_{l,i}}\right)$        **Communication**.        Transmit the intermediate $\varphi_{k,i}$ to all neighbors in $N_{k}$.        **Combination**.             $w_{k,i}=\sum_{l\in N_{k}}c_{lk}\varphi_{l,i}$              $N_{k}$ are the neighbor nodes of node *k* in the communication subnetwork.

## 4. Tracking Performance Analysis

The tracking performance of the proposed diffusion logarithm-correntropy algorithm is analyzed in this section. The convergence condition is first studied with ${\tilde{w}}_{i}$, defined as the error signal, which is a time-varying parameter under the random walk model:

(19) $${\tilde{w}}_{i}≜w_{i}^{*}-w_{k,i}.$$

It has been proven that subtracting $w_{i}^{*}$ from both sides of the update procedure on a node and then taking the expectation value leads to the following relation under stationary conditions in [10]:

(20) $$E{\tilde{w}}_{i}=\left[I-\mu \sum_{k=1}^{N_{k}}R_{u,k}\right]\;E\left\{{\tilde{w}}_{i-1}\right\}.$$

Then, considering the Assumptions 2 and 3 of the random sequence $\left\{\eta_{i}\right\}$, we observe that $w_{i}^{*}$ has a constant mean and hence $E\left[w_{i}^{*}\right]=E\left[w_{i-1}^{*}\right]$ under the relation in Equation (1). Taking the expectation value leads to the following relation under non-stationary conditions in Equation (1). We obtain

(21) $$E{\tilde{w}}_{i}=\left[I-\mu \sum_{k=1}^{N_{k}}R_{u,k}\right]\;E\left\{{\tilde{w}}_{i-1}+\eta_{i}\right\}.$$

In Equation (21), $\eta_{i}$ is a zero-mean variable sequence with covariance matrix $R_{\eta }$. Our purpose is to achieve mean square deviation (MSD) and excess mean square error (EMSE) for each node, which are defined as:

(22) $$MSD_{k}=E{∥{\tilde{w}}_{k,\infty }∥}_{I}^{2}.$$

(23) $$EMSE_{k}=E{∥{\tilde{w}}_{k,\infty }∥}_{R_{u,k}}^{2}.$$

In the proposed algorithm in Equation (17), the error signals can be defined as follows:

(24) $${\tilde{\psi }}_{k,i}≜w_{i}^{*}-\psi_{k,i},$$

(25) $$e_{k,i}≜d_{k,i}-u_{k,i}{\tilde{\psi }}_{k-1,i}.$$

In the non-stationary case with $w_{i}^{*}=w_{{}_{i-1}}^{*}+\eta_{i}$, based on the definition in Equation (15), $J_{k}^{local}\left(w\right)$ is twice continuous differentiable when w≠0. Then we obtain the Hessian matrix of $J_{k}^{local}\left(w\right)$, which is defined as $\nabla_{w}^{2}J_{k}^{local}\left(w\right)$.

From Lemma 1 and Theorem 1 in [12], the bound Hessian is: $\lambda_{k,\min}I_{M}\leq \nabla_{w}^{2}J_{k}^{local}\leq \lambda_{k,\max}I_{M}$ and $0\leq \lambda_{k,\min}\leq \lambda_{k,\max}$.

Equations (16)–(18) cause gradient error. The error recursion is then given by

(26) $${\tilde{\varphi }}_{k,i}=\left[I_{M}-\mu H_{k,i-1}\right]{\tilde{w}}_{k,i-1}-\mu n_{k,i},$$

(27) $${\tilde{w}}_{k,i}=\sum_{l\in N_{k}}c_{lk}{\tilde{\varphi }}_{l,i}.$$

In Equation (26), as a positive-definite random matrix, $H_{k,i-1}$ is defined as

(28) $$H_{k,i-1}≜\int_{0}^{1}\nabla_{w}^{2}J_{k}^{local}\left(w^{*}-t\cdot {\tilde{w}}_{k,i-1}\right)dt.$$

Applying Jensen’s inequality to Equation (27), the variance of ${\tilde{w}}_{k,i}$ is bounded by

(29) $$E{∥{\tilde{w}}_{k,i}∥}^{2}\leq \sum_{l\in N_{k}}c_{lk}E{∥{\tilde{\varphi }}_{l,i}∥}^{2},$$

where ${∥.∥}^{2}$ is a convex function and represents the squared Euclidean norm.

Integrating both sides of Equation (26), we achieve

(30) $$E{∥{\tilde{\varphi }}_{k,i}∥}^{2}=E\left({∥{\tilde{w}}_{k,i-1}∥}_{\Sigma_{k,i-1}}^{2}\right)+\mu^{2}E{∥n_{k,i}∥}^{2},$$

(31) $$\begin{array}{c} \Sigma_{k,i-1}≜{\left(I_{M}-\mu H_{k,i-1}\right)}^{T}\left(I_{M}-\mu H_{k,i-1}\right)+\mu H_{k,i-1}^{T}H_{k,i-1}. \end{array}$$

It follows from the bound Hessian and Equation (28) that

(32) $$0\leq \Sigma_{k,i-1}\leq \tau_{k}^{2}I_{M},$$

where

(33) $$\tau_{k}^{2}≜\max\left\{{\left(1-\mu_{k}\lambda_{k,\max}\left(R_{u,k}\right)\right)}^{2},{\left(1-\mu_{k}\lambda_{k,\min}\left(R_{u,k}\right)\right)}^{2}\right\}+\mu^{2}\lambda_{{}_{k,\max}}^{2}\left(R_{u,k}\right).$$

According to [12], substituting Equation (32) into Equation (30), we get

(34) $$E{∥{\tilde{\varphi }}_{k,i}∥}^{2}\leq \left(\tau_{k}^{2}+\alpha \mu_{k}^{2}\right)E{∥{\tilde{w}}_{k,i-1}∥}^{2}+\mu^{2}\sigma_{{}_{n,k}}^{2},$$

where α≥0 is a constant. The global MSD is introduced, which leads to

(35) $${\tilde{w}}_{i}≜col\left\{E{∥{\tilde{w}}_{1,i}∥}^{2},E{∥{\tilde{w}}_{2,i}∥}^{2},\cdots ,E{∥{\tilde{w}}_{N,i}∥}^{2}\right\}.$$

We collect the $\left\{c_{lk}\right\}$ into N×N matrices $C_{i}$ , such that $C=E\left[C_{i}\right]$. The $C_{i}$ is left-stochastic, that is, $C_{i}^{T}1_{N}=1_{N}$, $1_{N}$ means the N×1 all one vector. From Equations (29) and (35), it holds that

(36) $${\tilde{w}}_{i}\underset{-}{≺}C^{T}\Gamma {\tilde{w}}_{i-1}+C^{T}\Xi 1_{N},$$

where ⪯ denotes element-wise ordering and

(37) $$\Gamma ≜diag\left\{\tau_{1}^{2}+\alpha \mu^{2},\cdots \tau_{N}^{2}+\alpha \mu^{2}\right\},$$

(38) $$\Xi ≜diag\left\{\mu^{2}\sigma_{{}_{n,1}}^{2},\cdots ,\mu^{2}\sigma_{n,N}^{2}\right\}.$$

In order to ensure the stability of the proposed algorithm in the mean sense, according to Theorem 1 (mean−squarestability) in Reference [12], it should hold that

(39) $$\mu_{k}<\min\left\{\frac{2\lambda_{k,\max}^{}\left(R_{u,k}\right)}{\lambda_{k,max}^{2}\left(R_{u,k}\right)+\alpha },\frac{2\lambda_{k,\min}^{}\left(R_{u,k}\right)}{\lambda_{k,\min}^{2}\left(R_{u,k}\right)+\alpha }\right\}.$$

Since $\lambda_{k,\min}^{2}\left(R_{u,k}\right)+\lambda_{k,\max}^{2}\left(R_{u,k}\right)\geq \lambda_{k,\min}^{2}\left(R_{u,k}\right)$. As i→∞, which indicates

(40) $$\lim_{i\to \infty }\;{∥{\tilde{w}}_{i}∥}_{\infty }\leq \frac{{∥\Xi ∥}_{\infty }}{1-{∥\Gamma ∥}_{\infty }}=\frac{\max_{k}\left(\mu^{2}\sigma_{n,k}^{2}\right)}{1-\max_{k}\left(\tau_{k}^{2}+\alpha \mu^{2}\right)},$$

where ${∥.∥}_{\infty }$ is the $l_{\infty }$ norm. When the step-sizes $\left\{\mu \right\}$ are sufficiently small, we can further yield the conclusion that

(41) $$\lim_{i\to \infty }\;{∥\tilde{w}{}_{i}∥}_{\infty }\leq \frac{\sigma_{n,k}^{2}}{2\min_{1\leq k\leq N}\;\left(\lambda_{k,\min}\right)}\frac{\mu_{\max}^{2}}{\mu_{\min}}.$$

According to the bound in Equation (41), if step-sizes $\left\{\mu \right\}$ are sufficiently small, the MSD of each node is $E{∥{\tilde{\omega }}_{k}\left(i\right)∥}^{2}$, which can become sufficiently small.

## 5. Simulation Results

**Figure 3:**
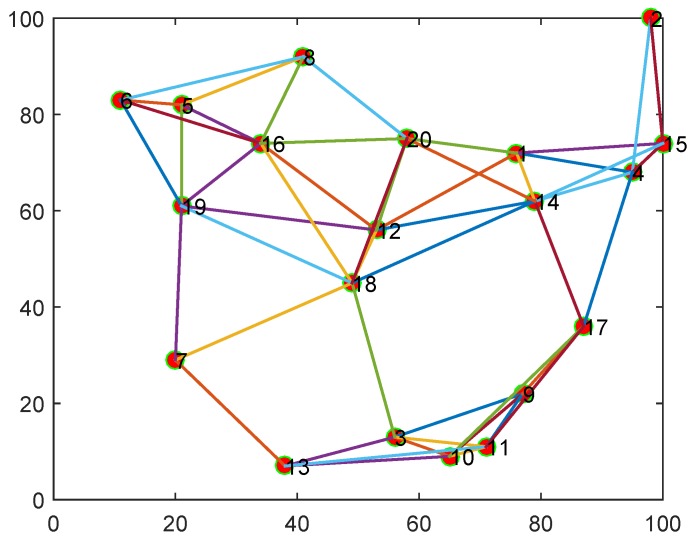
The topology of sensor network with 20 nodes.

In this section, to verify the performance of the proposed diffusion logarithm-correntropy algorithm, we considered a network consisting of 20 nodes and 50 communication links. The topology is shown in Figure 3. The sensor nodes were randomly deployed in an area of 100×100 and the communication distance between nodes was set as 35. All results below were averaged over 150 independent Monte Carlo simulations with randomly generated samples.

**Figure 4:**
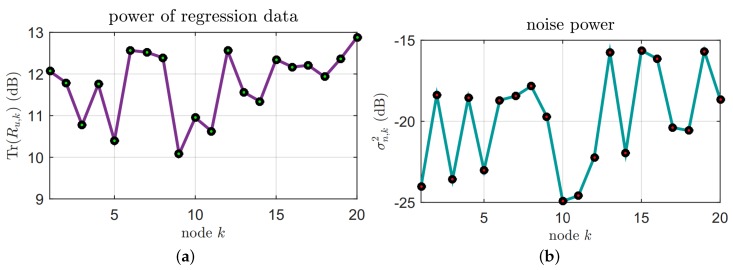
(**a**) Regressor statistics; (**b**) Noise variances.

**Figure 5:**
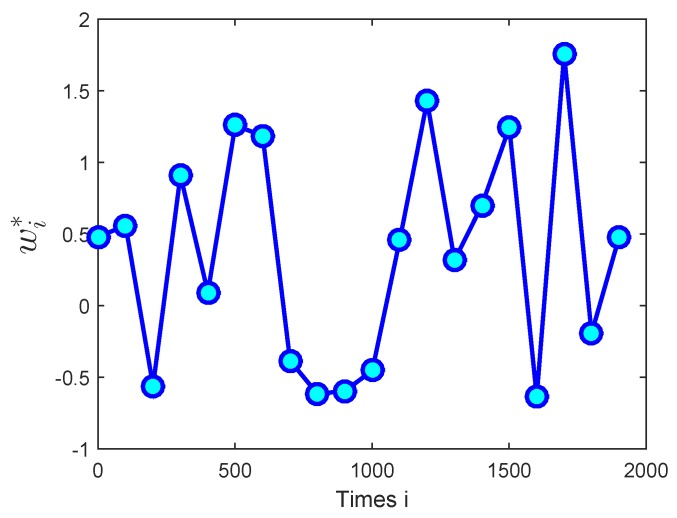
The desired time-varying vector, $w_{i}^{*}$.

In this simulation part, firstly, the performance of the proposed algorithm was verified in a non-stationary environment over sensor networks and the communication links were ideal. In Figure 4, the regression inputs $\left\{u_{k}\left(i\right)\right\}$ are independent identically distributed (i.i.d.), which are zero-mean Gaussian vectors with covariance matrices $R_{u,k}=\sigma_{u,k}^{2}I_{M}$, and the $\sigma_{u,k}^{2}$ is the input variance. The background noises $\left\{n_{k}\left(i\right)\right\}$ are drawn independently of the regressors and are i.i.d. The unknown parameter vector $w_{i}^{*}$ is time-varying, as Figure 5 shows. The fixed step-size μ = 0.002 is used in the simulations.

**Figure 6:**
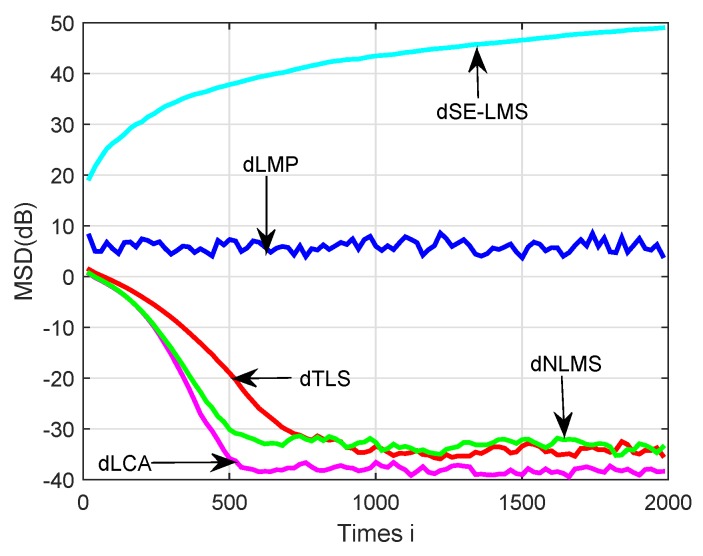
A comparison of simulated MSD learning curves in a non-stationary environment over sensor networks for the diffusion sign-error least-mean-square (dSE-LMS), diffusion minimum average p-power (dLMP), gradient-descent total least-squares (dTLS), diffusion normalized least-mean-square algorithm (dNLMS), and diffusion logarithm-correntropy algorithm (dLCA) algorithms.

The MSD learning curves are plotted in Figure 6. It shows that the proposed dLCA algorithm obtained the fastest convergence rate when compared with the dSE-LMS, dLMP, dTLS, and dNLMS algorithms. It also shows that the dLCA algorithm could achieve relatively good performance in terms of the network MSD. The proposed algorithm had relatively smaller MSD than the mentioned algorithms. From these simulation results, it can be seen that diffusion logarithm-correntropy algorithm exhibited better tracking ability in non-stationary environments than the existing classical algorithms.

**Figure 7:**
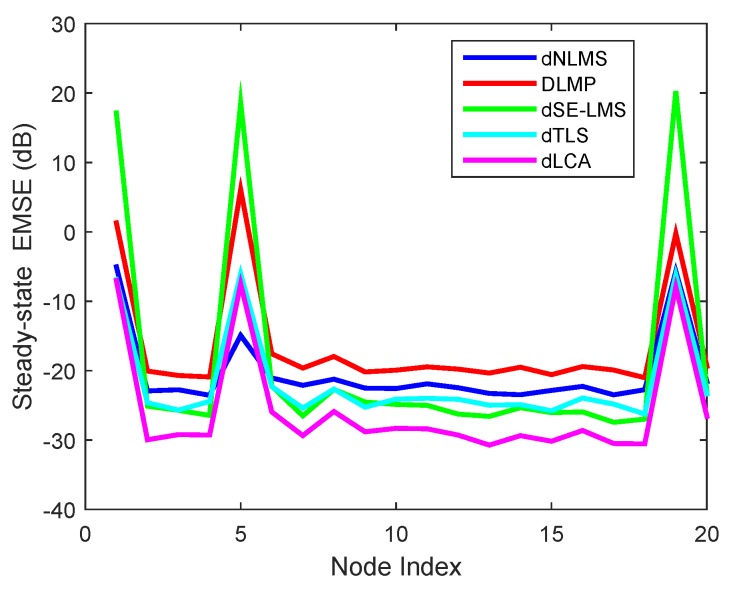
Estimated accuracy comparison in terms of excess mean-square error (EMSE) on each node for the dSE-LMS, dLMP, dTLS, dNLMS, and dLCA algorithms.

Figure 7 compares the steady-state EMSE performances of related algorithms on each node in the sensor networks. It can be observed that a large difference was observed at some nodes that achieved low EMSE. By averaging over 150 experiments and over 50 time samples after convergence, the steady-state EMSE values were obtained. The proposed algorithm captured a better trend of the steady-state performance than other algorithms.

**Figure 8:**
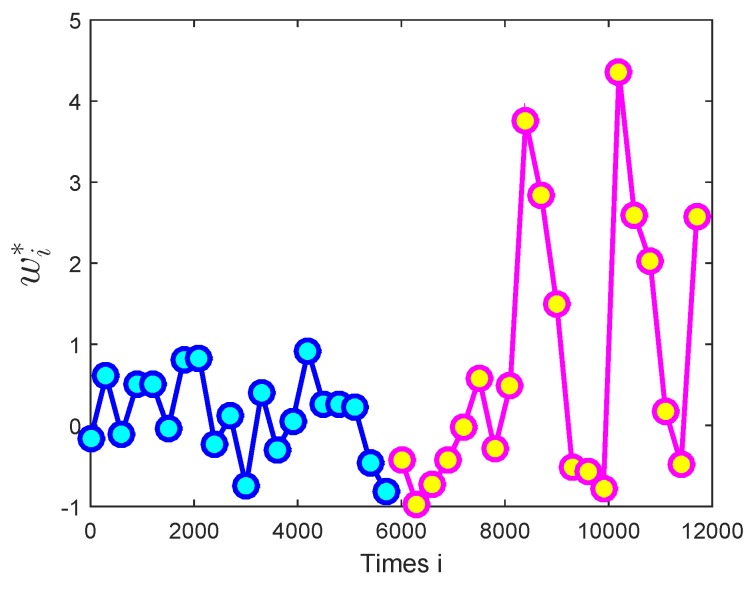
The desired time-varying vector with link changing, $w_{i}^{*}$.

Secondly, to further simulate the non-stationary scenarios in sensor networks, the link was assumed to change at time 4000. The unknown parameter vector $w_{i}^{*}$ was time-varying with link changing, as Figure 8 shows.

**Figure 9:**
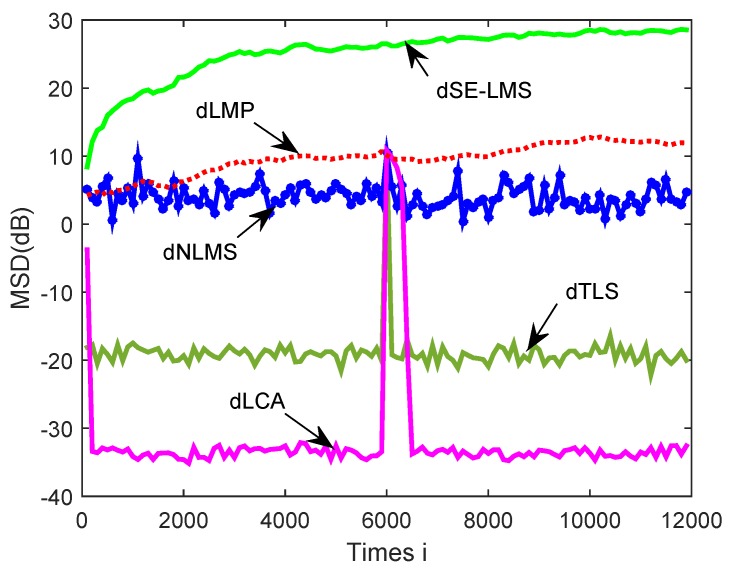
A comparison of simulated MSD learning curves of the global network for the dSE-LMS, dLMP, dTLS, dNLMS, and dLCA algorithms in non-stationary environments over sensor networks with links changing.

From the simulation results shown in Figure 9, in non-stationary environments over sensor networks with links changing, the diffusion logarithm-correntropy algorithm had smaller MSD than other related algorithms, such as dSE-LMS, dLMP, dTLS, and dNLMS algorithms. It further shows that the proposed dLCA algorithm had better tracking ability in non-stationary environments.

**Figure 10:**
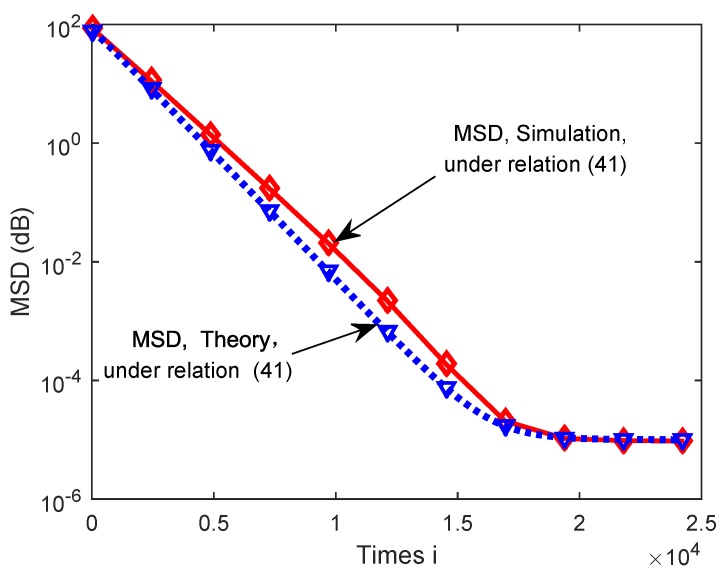
Theoretical and simulated MSD curves of the proposed dLCA algorithm under Equation (41).

Finally, we compared the simulated network MSD curves with theoretical results under Equation (41) in Figure 10. One can see that theoretical network MSD curves of the proposed algorithm showed good match with its simulated MSD curves.

## 6. Conclusions

To solve the problem of parameter estimation in non-stationary environments over sensor networks, each node in the sensor networks was equipped with the logarithm-correntropy cost function. The proposed algorithm can gradually adjust the cost function in its optimization based on the estimation error amount. We investigated the tracking behavior of the proposed algorithm under non-stationary conditions. Furthermore, the simulations were implemented in the non-stationary environments, where the parameters were time-varying with link changing. Simulation experiments were conducted to verify the analytical results, and illustrated that the proposed algorithm outperformed existing algorithms, such as dSE-LMS, dLMP, dTLS, and dNLMS algorithms.
